# Aggression against Nursing Personnel during the First Wave of COVID-19 Pandemic: An Internet-Based Survey

**DOI:** 10.3390/nursrep13040116

**Published:** 2023-10-07

**Authors:** Juan Pablo Sánchez-de la Cruz, Alma Delia Genis-Mendoza, María Lilia López-Narváez, Thelma Beatriz González-Castro, Isela Esther Juárez-Rojop, Carlos Alfonso Tovilla-Zárate, Humberto Nicolini

**Affiliations:** 1 División Académica Multidisciplinaria de Comalcalco, Universidad Juárez Autónoma de Tabasco, Comalcalco 86650, Mexico; jpsanchezc94@gmail.com (J.P.S.-d.l.C.); dralilialonar@yahoo.com.mx (M.L.L.-N.); 2 Servicio de Atención Psiquiátrica, Hospital Psiquiátrico Infantil Dr. Juan N. Navarro, Mexico City 14080, Mexico; adgenis@inmegen.gob.mx; 3 División Académica Multidisciplinaria de Jalpa de Méndez, Universidad Juárez Autónoma de Tabasco, Jalpa de Méndez 86250, Mexico; thelma.glez.castro@gmail.com; 4 División Académica de Ciencias de la Salud, Universidad Juárez Autónoma de Tabasco, Villahermosa 86100, Mexico; iselajuarezrojop@hotmail.com; 5 Laboratorio de Genómica de Enfermedades Psiquiátricas y Neurodegenerativas, Instituto Nacional de Medicina Genómica, Mexico City 14610, Mexico

**Keywords:** COVID-19, Latin America, aggression, victimization

## Abstract

(1) Background: health care workers, particularly nurses, have been regularly assaulted during the COVID-19 pandemic. Purpose: to evaluate the prevalence and location of assaults against nursing personnel in Latin America, and to determine predictor factors for aggression against nurses. (2) Methods: A cross-sectional online survey was answered by 374 nurses working in health care during the COVID-19 pandemic. The aggression against nurses was estimated using the Victimization Scale. (3) Results: A total of 288 nurses were included in this study. The victimization scale showed that 52.1% of nurses have suffered aggression by the general population during the COVID-19 pandemic. Males were more likely to be attacked than females (*p* < 0.05). Additionally, males were attacked more frequently on public transport (x^2^ = 6.72, *p* = 0.01). The home neighborhood and markets were other locations with a higher risk of being assaulted (OR: 3.39, CI: 1.53–7.50). (4) Conclusions: Our results indicate that nurses in Latin America who work during the COVID-19 pandemic and social isolation have been frequently assaulted by the general public. Males are more frequently attacked than females and the main places of aggression are public transportation, their home neighborhood and supermarkets. Implications for nursing practice: it is necessary to create and implement protocols and guidelines to support nursing personnel during the COVID-19 pandemic. This study was retrospectively registered at the Juarez Autonomous University of Tabasco (103/CIPDACS/2020) on the (08/2020).

## 1. Introduction

The new disease caused by the SARS-CoV-2 virus has been called COVID-19 [1,2]. The first cases of this disease was reported in China at the end of 2019 and, in a short time, it became a serious public health problem [3]. The rapid dispersion and accelerated growth of COVID-19 cases around the world prompted the World Health Organization to announce a state of sanitary emergency and to declare it a pandemic on 11 March 2020 [4,5]. 

In Latin America, the first case was reported in Brazil, on 25 February 2020 [6], followed by cases in Mexico, Ecuador and the Dominican Republic [7]. The first cases of COVID-19 in the region came from the European countries Italy and Spain [8]. Preventive measures have been implemented in Latin American countries; however, the current social, political and economic crises are problems that hinder the preparation of Latin American countries to face the COVID-19 pandemic [9].

In addition to fighting the health crisis that COVID-19 represents in the region, nursing personnel face a wave of attacks and discrimination for belonging to the health team [10]. A common idea at the beginning of the Ebola epidemic or the COVID-19 pandemic was the idea that health care workers were the ones who spread the virus. Then, health care workers became victims of attacks and social discrimination [11]. For instance, more than 200 attacks against health care workers were reported in Mexico since the start of the COVID-19 pandemic [12,13].

In Mexico, assaults toward health personnel are present in various regions of the country and in various forms. Insults, acts of rejection in their home neighborhood, the restriction of entrance to some establishments, being splashed with chlorine, being thrown hot drinks and blows that cause serious injuries have been common [14]. This situation is also present in other regions of Latin America; for example, discrimination in supermarkets has been reported in Colombia, assaults have been reported in Argentina and Bolivia in their home neighborhoods, and assaults frequently occur on public transport in Brazil [15]. This atmosphere of aggression against the health personnel is not unique to Latin America. The literature suggests that aggression was also observed to a lesser degree in some cities of first-world countries [16].

The aggressors justify their acts on the grounds that health personnel are responsible for the spread of the virus and consider them a source of contagion. It is not the first time that the health personnel have been subjected to regular assaults in Latin America [17]. These attacks have occurred within hospitals and in emergency rooms [18], with the nursing staff being the most affected [18,19,20].

Previous to the COVID-19 pandemic, in Latin America, it was estimated that 11.1% of health care workers had experienced physical violence; of those, 47.4% were nursing staff. In more detail, psychological violence was observed in 42.1% of health care workers, where 62.3% belong to nursing staff [21]. Moreover, violence and aggression against health care workers in Latin America increased during the COVID-19 pandemic, up to 54.8% just in the workplace [22]. Furthermore, the health personnel were also attacked outside their workplace [23]. Currently, violence against health care workers has increased, especially toward nursing workers [24], not only by the parents of the patients. Intra-hospital violence has increased [25]. Therefore, the objectives of this study were (1) to determine the prevalence and places of aggressions toward the nursing personnel using an internet-based survey through the victimization scale, and (2) to compare the sociodemographic characteristics and determine predictive factors for aggression in nurses in Latin America.

## 2. Materials and Methods

### 2.1. Ethics Statement

This study complies with the Declaration of Helsinki and was approved by a local ethics committee. Before starting the survey, the participants were explained the objectives of this study. Written informed consent to participate was not obtained but inferred through completion of the questionnaire.

### 2.2. Design of This Study

We conducted a cross-sectional, comparative, retrospective study, performed through an internet-based survey.

### 2.3. Participants

**Figure 1:**
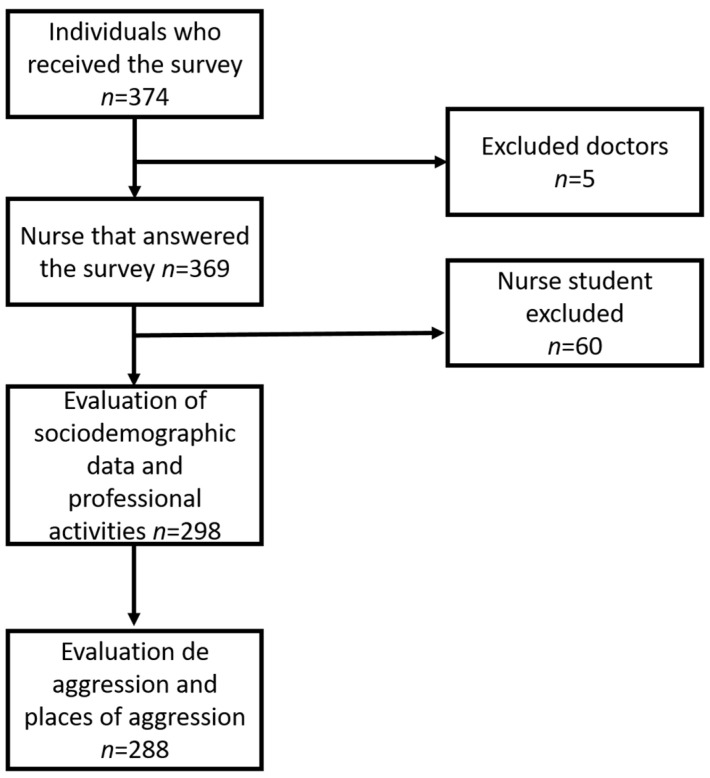
Flowchart of the stages of this study and the participants included.

The participants’ recruitment for the present study was performed using a non-probabilistic sampling. This study started with 374 individuals from Latin America (Figure 1).

### 2.4. Inclusion and Exclusion Criteria

Inclusion criteria: (a) Only nursing staff were allowed to participate, including interns (students in training), nursing assistants (technician), general and specialist nurses. (b) Nursing staff working in health care. (c) Not having COVID-19 diagnosis at the time of this study nor positive to PCR test for COVID-19. (d) Working in a Spanish-speaking country of Latin America. 

Exclusion criteria: (a) students and (b) being positive to COVID-19 at the time of this study. Finally, participants who did not fully answer the survey were also excluded.

### 2.5. Mesurment Procedure

We designed a survey entitled “Aggression against nursing due to COVID-19”. We conducted the survey from 29 April to 25 May 2020. The survey was designed in SurveyMonkey [26].

The survey included four sections. The first one was designed to collect sociodemographic data of the participants (including gender, age, school level, nurse profile and marital status). The second section collected information regarding professional activities and characteristics associated with their place of work (level of health care and working hours), as well as if the nurse was working on the frontline treating individuals with COVID. In the third section, we collected information regarding aggression against nursing staff. We used the validated Victimization Scale [27]. This scale measures the frequency of being victimized during the past seven days prior to the survey [28]. It comprises 10 self-reported items, and each item represents one instance of victimization reported. The score indicates the number of times that other individuals assaulted the participants in the previous week. The total is scored by adding the responses; however, we took a response of one or higher as positive to victimization. The Victimization Scale has been adapted into Spanish and validated in nursing students with adequate internal consistency (Cronbach’s alpha = 0.85) [27,29]. Fourth, we enquired about the places where nurses had been victims of aggression; we considered where news and newspapers had reported aggression against health personnel (on the streets, in my home neighborhood, condominium, my community, in my workplace, in markets, in public transport, and I have been denied access to certain establishments).

### 2.6. Recruitment

Nurses were invited to participate in chatrooms on social media. The invitation included the objectives of this study and ended with a link to survey. Many chatrooms on social media (Facebook) in Spanish were used to disseminate the invitation, and these included “Enfermeros Argentinos Unidos”, “Enfermeras de Chile”, “Enfermeras de Honduras”, “Enfermeros de Puerto Rico”, “Solo Médicos”, “Enfermeras y Paramédicos de México” and others. To protect the identity of the participants, no personal information was collected. Only Latin American countries represented in the “Asociación Latinoamericana de Escuelas y Facultades de Enfermería ALADEFE” (in Spanish) were included in this study.

The final stage was the data collection. Then, the information was downloaded in Excel format. Finally, we checked repetitions of IP addresses to avoid duplications.

### 2.7. Statistical Analysis

Descriptive statistics were used. Frequencies and percentages are used for categorical variables, with mean and standard deviations for continuous variables. To evaluate differences by sex, for categorical variables, the contingency table x^2^ test was performed; for continuous variables, we used *t*-test. Also, we performed two logistic regression analyses. First, to calculate the probability of aggression to nurse staff, as explanatory variables, we included sex, age, marital status, nurse profile, the level of health care they work at and if they worked directly in the COVID-19 area. By contrast, nursing staff that suffered aggression was used as the outcome variable. Second, we estimated the odds ratio of the place where the aggression took place. In this model, we included the places of the aggression as explanatory variables and nurses who suffered from aggression as an outcome variable. Then, regression coefficients (*β*), odds ratios and 95% confidence intervals were calculated. Significance was established at *p* < 0.05.

## 3. Results

### 3.1. Demograohic Characteristics

This survey was conducted on an online platform; initially, 374 individuals of Latin-American countries participated. Nonetheless, only 288 individuals completed all the phases of this study and were included (Figure 1). The majority of participants were individuals from Mexico (*n* = 190, 66.0%), followed by individuals from Argentina and Colombia (*n* = 16, 5.6%), Costa Rica (*n* = 14, 4.9%), Honduras (*n* = 12, 4.2%), Chile (*n* = 10, 3.5%), Peru (*n* = 9; 3.1%) and El Salvador (*n* = 7, 2.4%), while Bolivia, Cuba, Ecuador, Puerto Rico and Venezuela accounted for <11% of the sample. The sociodemographic characteristics are shown in Table 1. The majority of participants who answered the survey were females (78.1%, *n* = 225). The mean age was 35.36 ± 10.47 ranging from 18 to 63 years old. The majority of participants had a nursing degree (51.7%, *n* = 149). The participants were working mainly at secondary health-care level (45.8%, *n* = 132). They were working between 6 and 8 h a day (58.3%, *n* = 168). The majority had been working on the frontline of defense against COVID-19 (*n* = 223, 77.4%).

The comparisons of demographic features between male and female nurses are shown in Table 1. Statistical differences emerged between groups in terms of working on the front line of COVID-19. The proportion of males working on the front line of defense against COVID-19 was larger than the proportion of females (x^2^ = 4.49, *p* = 0.03).

### 3.2. Victimization and Places of Aggression against Nurses

Table 2 displays the comparative analysis of nurses who were victimized during the seven days prior to the survey. We observed that the majority of nurses had been victimized (*n* = 150, 52.1%) during this time. Proportionally, significant differences emerged by sex, as males received more aggression than women. The aggressions reported included the following: an individual teased me to make me angry, an individual said things about me to make others laugh, an individual encouraged me to fight, an individual called me (or my family) bad names, and an individual threatened to hurt me or to hit me (*p* < 0.05) (Table 2). Next, we analyzed differences by sex according to the spot where the aggression took place. The places of aggression against nurses were similar between males and females; however, male nurses were more frequently victimized on public transport than females (x^2^ = 6.72, *p* = 0.01).

### 3.3. Logistic Regression Analysis

The logistic regression models included the following characteristics: sex, age, marital status, nurse profile, level of health care that the nurses were working at, number of hours working per day, working on the front line against COVID-19 and spots where aggressions took place. For the prediction analysis of victimization of nurses, the logistic regression model classified 59.1 of victimized nurses and 78.3 of not victimized nurses as controls. The model showed a valid construct according to the Hosmer and Lemeshow test (*p* = 0.37). We found that being a male (OR: 1.94, CI: 1.02–3.67); in my home neighborhood, condominium, community (OR: 3.39, CI: 1.53–7.50); and inside markets were predictors for being victimized (Table 3).

## 4. Discussion

The objectives of our research were to determine the prevalence and place of aggression toward nursing personnel in Latin America during the COVID-19 pandemic, using the Victimization Scale, as well as to compare the sociodemographic characteristics of aggression in nursing personnel in Latin America and to determine predictive factors for aggression in our region. To our knowledge, this is the first study that evaluates these variables in Latin American countries in the context of the COVID-19 pandemic.

First, our results showed that 52.1% of nursing personnel in Latin America had been victimized during the COVID-19 pandemic, which means that more than half of the participants were victims of aggression during the 7 days prior to the survey. Workplace aggressions toward the health personnel is a phenomenon that has been described prior to the COVID-19 pandemic in many countries worldwide [30,31,32]. A recent meta-analysis showed a prevalence of 62.4% of aggression against health professionals [33]; more precisely, the lowest was 40.8% in Spain [34,35] and the highest was 94.1% in Germany [36].

A different scenario has been observed during the COVID-19 pandemic. For example, during isolation in Italy, people at home applauded for the efforts made by the health personnel. The United Kingdom started the “clap for our carers” campaign [37], a behavior that spread to other countries including The United States, France, India and Turkey [38]. In China, using scientific publications, a call was made to respect, honor and applaud all the health personnel who faced the outbreak of the epidemic [39]. Contrarily, in Latin America, many reports of assaults against health professionals and particularly against nurses were made. Initially, this behavior was observed in Mexico [12], where a total of 53 attacks were reported in the first two months of the epidemic in the country.

The most serious cases of aggression had been the murder of a pediatric nurse using a firearm [40] and the violent murder of two nurses and a secretariat who worked at the Mexican Social Security Institute [41]. Along with the development of the pandemic in the Latin American region, more countries reported acts of aggression and discrimination against medical personnel, with nurses being most affected. It is not possible to know the true reasons that motivate the general public to attack nursing personnel during the COVID-19 pandemic. However, some journalistic articles mentioned that aggression is the stigma and that nursing personnel are carriers of the virus and are responsible for its spread [14].

Some possible factors could be associated with the link between aggression and occupation. How can the perpetrator identify the nursing staff? In Mexico, it is common for health personnel to wear a uniform. Health care students and doctors wear work uniforms even outside their workplace. Nursing staff wear their uniform from home to work or to carry out daily activities, like going to the supermarket. For their part, medical staff wear a white gown, which is also used outside the hospital system. On the other hand, in Latin America, for example, Colombia, the use of surgical gowns is widely used, either with the identification of the health institution or the medical school. Therefore, anywhere outside the health system, it is easy to identify health personnel.

Second, we found statistically significant differences by sex, as men are more frequently attacked than women. The same had been reported previous to the coronavirus pandemic [33]. No statistically significant difference was observed when comparing aggressions toward nurses who work on the front line of defense against COVID-19 and those who do not work on the front line against COVID-19, which means that the aggression against the nursing personnel is independent of their area of work. This suggests that the aggression is associated with the idea that health care workers are who spread the virus, more than the attention in the service of health. Interestingly, the level of health care and the nurses’ profile were not related to the aggressions. These results show the need that in a new epidemic or pandemic, the news and social media need help to educate the general population and make them understand that health care workers do not spread the virus.

We further analyzed the differences by gender and places where aggressions occurred. Our findings indicate that places of aggression against nursing personnel were similar between males and females; however, aggression on public transport was more frequently observed in male nurses. The attacks reported before the COVID-19 pandemic mainly occurred within public hospitals, health centers, doctors’ offices, etc., that is, on the job sites [30,32,42]. In contrast, during the COVID-19 pandemic in Latin America, attacks mainly occur on the streets [43], public transportation [44] and home neighborhoods [45].

In the context of patient care, a climate of aggressiveness can also be observed. Recommendations have been issued and action protocols have been created to reduce the incidence of assaults [46]. These include preventive measures such as changes in the infrastructure of health care units, more space in waiting rooms, the creation of new exits and escape routes, the installation of surveillance cameras and more security [47,48]. When and if there are aggression incidents, essential communication techniques have been recommended in order to remain calm, seek to neutralize the conflict, seek personal safety by evacuating facilities and activating emergency protocols [46]. After an incident, it is important to avoid violence and make a full report with the appropriate authorities [47]. These recommendations help to decrease the number of assaults and their severity, but do not prevent them completely. It is important to underline that these recommendations are of no use during the COVID-19 pandemic that we face currently, since most of the attacks occur outside hospitals and workplaces.

The consequences of those assaults toward nursing personnel vary depending on the type and severity of the aggression. For example, the impact of verbal aggression can have an important role on the mental health of nursing personnel, which is already affected by the stress caused by the health crisis, and may contribute to increasing psychosocial risk [13]. One study indicates that health professionals exposed to aggression have higher levels of anxiety, emotional exhaustion and burnout syndrome compared to those not exposed to aggression [49]. Constant verbal aggression can cause greater emotional exhaustion than physical aggression. At the same time, up to a third of those who have been attacked can trigger an episode of emotional stress [36], affecting their concentration and their performance at work, while physical assaults can cause injuries that could incapacitate the nursing staff. The health deterioration of nursing professionals represents a serious problem; it interferes with their performance, work activities, the quality of the service and the care provided.

Given the large number of attacks against nursing personnel in Latin America during the COVID-19 pandemic, the following recommendations are issued: (1) Nursing personnel should avoid wearing hospital or institutional uniforms when leaving their workplaces or in public places; changing their clothing may be a strategy that prevents them from being identified as health personnel in public areas. (2) Institutions must provide conflict mitigation techniques, communication techniques and body language reading; the objective is to develop skills to identify possible aggressors. (3) Mental health support should be provided to reduce the burden of work stress, which will allow the nursing staff to act more serenely/calmly in cases of being assaulted. (4) The medical institutions must establish protocols for situations of aggression within the work centers, increase security and request the identification of each person who enters the health establishments. (5) The foregoing should guarantee that reports of attacks are made to the relevant authorities. (6) Latin American countries should create new public policies that guarantee safety and protection for nursing and health personnel in situations of aggression. Additionally, the psychiatric and psychological support is necessary in the individual that has been attacked [50].

Some limitations can be observed in our study. First, our study evaluates the characteristics of aggression toward nursing personnel, but we did not evaluate the characteristics of the aggressors. Therefore, it is not possible to know the reasons why the general public attack nursing personnel. Second, the sample size is small. Although our survey was broadcasted on various social networks and nurses’ chatrooms, it was not answered in all Latin American countries. Also, a large number of participants did not complete the survey. Therefore, our sample is small and does not represent all the countries in Latin America. Despite these limitations, a strength of our study is that aggression was measured using a valid scale and, during the last 7 days prior to application, this differs from most studies that indicate aggression as a self-report and in unadjusted periods of time. Our study is an initial contribution on the attacks suffered by Latino nursing personnel during the COVID-19 pandemic. Although the COVID-19 pandemic is over, reports of violence against nursing staff continue [51]. Studies report violence against nursing staff mainly within the hospital institution [52]. It is noteworthy that the violence reported in this study was outside the health institution. Finally, the literature reports that the number of events of aggression against nursing staff is similar with and without the COVID-19 pandemic. However, more studies are required to understand the phenomenon more clearly in order to establish appropriate recommendations.

## 5. Conclusions

In conclusion, in our sample, the phenomenon of aggression toward nursing personnel during the COVID-19 pandemic exceeded 50%. Male nurses are more likely to be attacked than women; the aggressions do not depend on the schooling level or degree of the nursing staff, nor if they work on the front line against COVID-19. We identified that the most frequent site of aggression is public transport. Finally, male nurses who use public transportation have an increased risk of suffering an aggression, as well as in their home neighborhood, condominium and supermarkets. The causes of the assaults are unknown and have not been explained. Health care institutions in Latin America need to establish protocols and guidelines to support nursing personnel who have been attacked.

## Figures and Tables

**Table 1:** Sociodemographic characteristics of the participants.

Characteristics	Total (*n* = 288)		Males (*n* = 63)		Females (*n* = 225)			
	Number	Percentage	Number	Percentage	Number	Percentage	Statistics	
Sex			63	21.9	225	78.1		
Age; mean, SD	35.36 (10.47)		34.06 (10.77)		35.72 (10.37)		*t* = −1.08	*p* = 0.27
Marital Status								
Married	140	48.6	27	42.9	113	50.2	X^2^ = 7.45	*p* = 0.59
Single	125	43.4	35	55.6	90	40.0		
Divorced	21	7.3	1	1.6	20	8.9		
Widowed	2	0.7	0	0	2	0.9		
Nurse Profile								
Nurses in training	20	6.9	6	9.5	14	6.2	X^2^ = 3.86	*p* = 0.27
Nursing Assistant (Technician)	46	16.0	13	20.6	33	14.7		
General Nurse (degree)	149	51.7	33	52.4	116	51.6		
Specialized nurse	73	25.3	11	17.5	62	27.6		
What level of health care do you work at?								
First Level	79	27.4	20	31.7	59	26.2	X^2^ = 2.53	*p* = 0.28
Second Level	132	45.8	31	49.2	101	44.9		
Third Level	77	26.7	12	19.0	65	28.9		
Hours of work (type of working day/on call)/day								
4–6 h	20	6.9	3	4.8	17	7.6	X^2^ = 0.62	*p* = 0.73
6–8 h	168	58.3	37	58.7	131	58.2		
8–12 h	100	34.7	23	36.5	77	34.2		
Working on the front line of defense against COVID-19								
Yes	223	77.4	55	87.3	168	74.7	**X^2^ = 4.49**	***p* = 0.03**
No	65	22.6	8	12.7	57	25.3		

Statistical significance in bold.

**Table 2:** Dimension of Victimization Scale by sex and location where the aggression took place.

	Total (*n* = 288)		Males (*n* = 63)		Females (*n* = 225)		Statistics	
Characteristic	Number	Percentage	Number	Percentage	Number	Percentage	X^2^	*p*-Value
*Victimization Scale*								
Positive to victimization	150	52.1	39	61.9	111	49.3	3.11	0.07
An individual teased me to make me angry	113	39.2	34	54.0	79	35.1	**7.34**	**0.007**
An individual beat me up	0							
An individual said things about me to make other individuals laugh (made fun of me)	72	25.0	25	39.7	47	20.9	**9.27**	**0.002**
An individual encouraged me to fight	20	6.9	8	12.7	12	5.3	**4.13**	**0.04**
An individual pushed or shoved me	24	8.3	9	14.3	15	6.7	3.74	0.05
An individual asked me to fight	15	5.2	7	11.1	8	3.6	**5.69**	**0.01**
An individual slapped or kicked me	1	0.3	0	0	1	0.4	0.28	0.59
An individual called me (or my family) bad names	65	22.6	21	33.3	44	19.6	**5.34**	**0.02**
An individual threatened to hurt me or to hit me	24	8.3	10	15.9	14	6.2	**6.01**	**0.01**
An individual tried to hurt my feelings	95	33.0	25	39.7	70	31.1	1.63	0.20
*Place where the aggression took place*								
On the street	35	12.2	11	17.5	24	10.7	2.12	0.14
In my home neighborhood, condominium, my community	38	13.2	7	11.1	31	13.8	0.30	0.58
In my workplace	20	6.9	5	7.9	15	6.7	0.12	0.72
In markets	46	16.0	10	15.9	36	16.0	**0.001**	**0.98**
In public transport	20	6.9	9	14.3	11	4.9	**6.72**	**0.01**
I have been denied access to certain establishments	4	1.4	1	1.6	3	1.3	**0.02**	**0.87**

Statistical significance in bold.

**Table 3:** Logistic regression model for the prediction of aggression to nurse staff.

	β	OR	95% CI	*p*-Value
**Aggression to nursing staff**				
Sex-Male	**0.66**	**1.94**	**1.02–3.67**	**0.04**
Age	−0.001	0.99	0.97–1.02	0.93
Marital Status	−0.30	0.73	0.49–1.11	0.14
Nurse Profile	0.09	1.09	0.75–1.60	0.62
What level of health care do you work at?	0.07	1.07	0.73–1.57	0.69
Work in COVID-19 area	0.07	1.08	0.56–2.06	0.81
**Place of the aggression**				
On the streets	1.06	2.90	1.21–6.91	0.16
In my neighborhood, condominium, community	**1.22**	**3.39**	**1.53–7.50**	**0.003**
In markets	**2.72**	**15.18**	**5.10–45.15**	**<0.001**

CI: confidence intervals, OR: odds ratio. Statistical significance in bold.

## Data Availability

Data are available under request.

## Public Involvement Statement

No public involvement in any aspect of this research.

## Guidelines and Standards Statement

This manuscript was drafted against the Strengthening the reporting of observational studies in Epidemiology (STROBE).
